# GCC2011 – 25 years of computational chemistry meetings

**DOI:** 10.1186/1758-2946-4-S1-A1

**Published:** 2012-05-01

**Authors:** Frank Oellien, Uli Fechner, Thomas Engel

**Affiliations:** 1 GDCh-CIC Division Chair, Intervet Innovation GmbH – MSD Animal Health, Zur Propstei, 55270 Schwabenheim, Germany; 2 GDCh-CIC Division Board Member, Beilstein-Institut zur Förderung der Chemischen Wissenschaften, Trakehner Str. 7-9, 60487 Frankfurt, Germany; 3 GDCh-CIC Division Co-Chair and Conference Chair, Fakultät für Chemie und Pharmazie, Universität München, Butenandtstr. 5-13, 81377 München, Germany

## 

25 years ago members of the Chemistry-Information-Computers (CIC) division of the German Chemical Society (GDCh) [1] realized that the usage of computers will play a major role in the processing of chemical information and that computational methods will have a large impact on chemical research approaches. At that time, Computational Chemistry was not yet established as a research field. Thus, scientists working in this area were typically chemists from all realms of chemistry who happened to have an interest in computers. To address the initial issues in the field of Computational Chemistry in a collaborative manner, the workshop *“Software-Entwicklung in der Chemie”* (software development in chemistry) — later renamed the CIC-Workshop — was established. The foundations were laid in this annual workshop and many projects and scientific outcomes originated from it in the years thereafter. While the initial workshops focused on the implementation of chemical databases, other topics, such as structure elucidation, structure representation and data mining, gained importance over the following years. The scientific network became more and more international over the intervening years, so much so that the CIC board decided in 2005 to change the, up to that point, German workshop into an international conference. The **7^th^ German Conference on Chemoinformatics** (GCC2011) was held from the 6^th^ to the 8^th^ of November 2011 in Goslar, Germany. The CIC division invited the chemoinformatics and molecular modeling community to the GCC2011 to celebrate the 25^th^ anniversary of the CIC-workshop.

The conference focused on recent developments and trends in the fields of

Chemoinformatics and Drug Discovery

*Chemical Information*, *Patents and Databases*

Molecular Modeling

Computational Materials Science and Nanotechnology

As always, contributions from other research areas of Computational Chemistry were also welcome.

**Figure 1:**
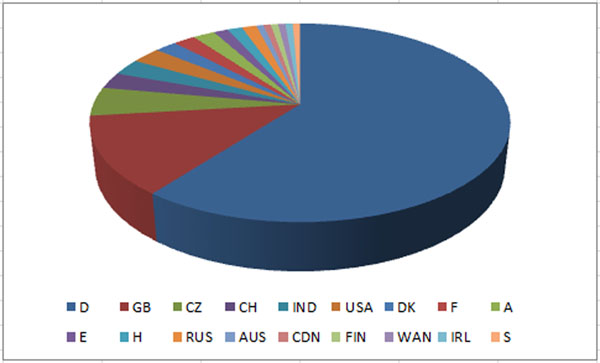
149 participants from 18 countries and 5 continents attended the GCC2011.

**Figure 2:**
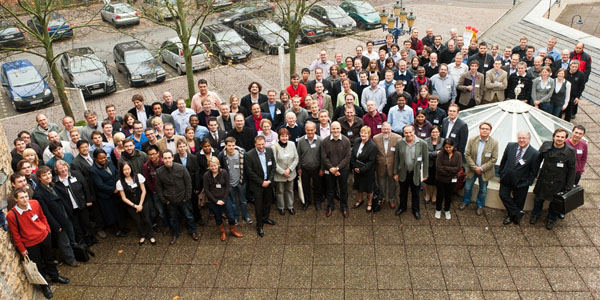
Participants of the 7^th^ German Conference on Chemoinformatics (GCC2011), November 6 – 8, 2011 in Goslar, Germany.

Despite the recent major changes in the pharmaceutical industry and the resulting decrease in research, the number of participants was comparable to the German Conference on Chemoinformatics in 2010. The international character of the conference was even more pronounced than in the preceding years due to the 149 participants from 18 countries (Figure 1, 2).

Following the tradition, the conference was opened up by the *“Free-Software-Session”* and the *“Chemoinformatics Market Place”* on Sunday afternoon. Four Open Source projects — Travis, Knime, ParadocS and DebiChem — were presented in the *“Free-Software-Session”* and three preconference workshops were given by the companies Chemical Computing Group, Tripos and Xemistry. The first day of the conference was concluded by dinner and an evening lecture by Johann Gasteiger (*“25 Years of CIC – Achievements and Future Goals”*) both of which took place in the ore mine of Rammelsberg.

The program of the following two days included plenary lectures from six well-known keynote speakers from industry and academia (Oliver Kohlbacher, University of Tübingen, Germany; Colin Groom, CCDC, Cambridge, UK; Eva Rauls, University of Paderborn, Germany; Colin Batchelor, RCS, Cambridge, UK; Herbert Köppen, Boehringer Ingelheim, Germany and Xavier Barril, University of Barcelona, Spain), as well as 17 lectures and 71 poster presentations.

**Figure 3:**
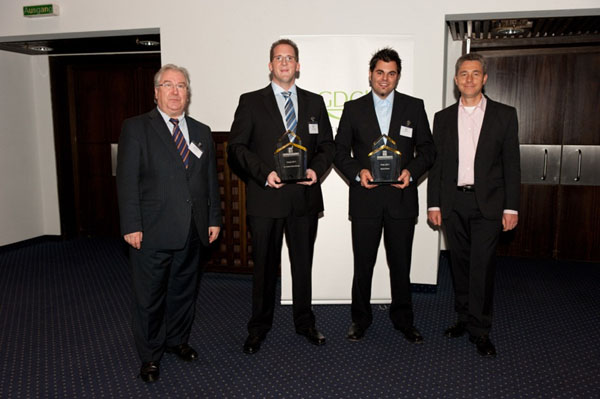
FIZ CHEMIE Berlin Awards 2011: from left to right, Rene de Planque (Head of the FIZ CHEMIE Berlin), Volker Hähnke (FIZ CHEMIE Berlin awardee, dissertation prize; NCBI, Bethesda, USA), Daniel Moser (FIZ CHEMIE Berlin awardee, master thesis prize; University of Frankfurt, Germany) and Frank Oellien (Chair of the GDCh CIC division).

A special highlight of the conference was the FIZ-CHEMIE-Berlin awards (Figure 3). The CIC division awards this prize every year to the best diploma thesis and the best PhD thesis in the field of Computational Chemistry. The prize for the PhD thesis was awarded to Dr. Volker Hähnke from the group of Prof. Gisbert Schneider, ETH Zurich for his dissertation *“Text-based Similarity Searching for Hit- and Lead-Candidate Identification”*. The award for the best diploma thesis was given to Daniel Moser from the group of Jun. Prof. Eugen Proschak, University of Frankfurt for his excellent master thesis “*Design of Dual Ligands Using Excessive Pharmacophore Query Alignment*”. Due to the closedown of the FIZ CHEMIE Berlin, these prizes were awarded for the last time in 2011. To continue its support of young German scientists in the future, starting with the GCC2012, the CIC division will endow the ***“CIC Advancement Award for Computational Chemistry”***.

The conference ended on Tuesday evening with a conference dinner and a speaker who was announced as a “special guest from MIT”. The special guest turned out to be Thomas Fraps, a magician who presented a very entertaining show. In the end he left a pleased and puzzled audience who could not find the answers to his “scientific experiments”. A lot of problems in Computational Chemistry have been solved in the last 25 years, yet the road ahead is full of challenging issues waiting to be tackled.
